# Research challenges in evaluating gender-based violence response services in a refugee camp

**DOI:** 10.1080/16549716.2020.1820713

**Published:** 2020-10-05

**Authors:** Alys McAlpine, Loraine J. Bacchus, Sheru W. Muuo, Stella K. Muthuri, Martin Bangha, Chimaraoke Izugbara, Giorgia Franchi, Tim Hess, Jo Spangaro, Rachel Pearson, Mazeda Hossain

**Affiliations:** a Department of Global Health and Development, London School of Hygiene and Tropical Medicine, London, UK; b Population Dynamics and Reproductive Health Program, African Population and Health Research Center, Nairobi, Kenya; c Violence Prevention and Response Unit, International Rescue Committee, London, UK; d School of Health and Society, University of Wollongong, Wollongong, Australia; e Population, Policy and Practice Research and Teaching Department, UCL Great Ormond Street Institute of Child Health, University of Bath, Bath, UK; f Centre for Women, Peace & Security, London School of Economics, London, UK

**Keywords:** Gender-based violence, refugee, evaluation, methodology, mixed-methods

## Abstract

This article presents a case study of research in Dadaab, Kenya to highlight some of the relevant challenges encountered while conducting gender-based violence research in humanitarian settings. A longitudinal mixed-methods design was used to evaluate a comprehensive case-management intervention in the refugee complex near the border of Kenya and Somalia. We present an overview of both expected and unexpected challenges during preparation and implementation of the research, adaptations made to the research design, and lessons learned for future research in similar contexts. Some of the key challenges were attributed to the highly securitized and remote environment of Dadaab refugee camp, like many refugee camp settings, which created limitations for sampling designs, interview locations, and also created particular burdens for the research team members conducting interviews. In addition to the camp environment, the dynamic nature of events and trends in the camp setting created barriers to follow-up with longitudinal cohort participants as well as uncertainty on how to plan for future implementation of research design phases in response to camp changes. Conducting research in humanitarian settings requires a flexible approach to accommodate the challenges that can impact both service delivery and research activities. The discussion presented in this article contributes to the evolving practical guidance on conducting research in humanitarian settings.

## Background

Humanitarian practitioners, donors and researchers concur that timely, rigorous research in emergency settings is critical for informed decision making. This primarily includes decision making on how to efficiently deliver the most effective interventions, both prevention and response, under acute resource and time constraints [1]. Furthermore, the pressing need to take innovation of humanitarian interventions to scale is often thwarted by insufficient evidence on the impact of these innovative responses in these settings.

In an attempt to address the challenge of humanitarian setting research, various groups such as the Humanitarian Innovation Fund (Elhra) have commissioned a number of reports synthesizing the challenges, lessons, and guidance on implementing needed rigorous informative humanitarian research [2]. This includes research guidance developed by other humanitarian actors, such as the Humanitarian Cluster and Area of Responsibility (AoR) specific research guidance, including research recommendations on Gender Based Violence (GBV) in humanitarian settings [3]. Other academic researchers with the help of expert informant groups have also developed guidance on research, monitoring and evaluation among conflict-affected populations [4]. The guidelines offer in-depth synthesis and reviews of what research methods have been used in these contexts and speak broadly to the unique challenges to empirical research in humanitarian settings, including intervention evaluation, but there remains a missed opportunity to regularly and openly share research lessons from individual studies and settings.

This article aims to address this gap and present a case country study to highlight some of the relevant challenges faced by our research team during the implementation of a longitudinal mixed-methods evaluation in the Dadaab refugee complex near the border of Kenya and Somalia. We aim to present an overview of our experiences and lessons learned while implementing research in a refugee camp context in order to inform other research partnerships in similar settings. This article does not aim to be exhaustive in addressing the challenges of GBV research in humanitarian settings, only specific lessons learned in relation to this study.

## Overview of the GBV intervention and the research in Dadaab

A range of GBV prevention and response activities, recommended by the global humanitarian community, have been implemented in the Dadaab refugee camps. However, similarly to many humanitarian settings, there was limited evidence on the effectiveness of GBV prevention and response strategies and interventions in Dadaab prior to this study. To address this evidence gap, from 2014–2017 the London School of Hygiene and Tropical Medicine (LSHTM) and the African Population and Health Research Center (APHRC) conducted research on an innovative GBV comprehensive case management model implemented in Dadaab which included a task-sharing approach with trained refugee community workers. As described in the full research report, refugee community workers are female and male refugees who are trained and employed via Dadaab’s incentive worker programme. Specific tasks are shared with the refugee community workers so that together the team can provide a tailored package of care to women and adolescent girls accessing their services, as well as GBV outreach and community mobilisation activities within the community [5]. This programme was being implemented in Dadaab by two humanitarian organisations – the International Rescue Committee (IRC) and Cooperative for Assistance and Relief Everywhere (CARE). The GBV comprehensive case management intervention is delivered within support centres and aims to be a one-stop shop model for survivors of GBV to confidentially access the range of services they may need. This includes professional psychosocial support, and medical services, collaboratively preparing a long-term case management plan, regular follow-up, and referral to any other support services needed that might be relevant to the client’s case but outside of the lead GBV response organisation’s service provision. This research was part of the *What Works to Prevent Violence Against Women and Girls* research programme funded by the UK Government’s Department for International Development (DfID). The complete findings from this study can be found in the full report [5].

### Evaluation aim and questions

The evaluation aimed to understand how the GBV response model of comprehensive case management with task sharing works to influence access to care, wellbeing, and health and safety among GBV survivors in the Dadaab refugee camps. The findings will be used to strengthen GBV services provided by humanitarian agencies in Dadaab and other contexts.

Within this aim we sought to address the following research questions:

1. What is the context of GBV in the Dadaab refugee camps?
2. What are the roles and experiences of IRC and CARE national staff and refugee community workers who deliver GBV response services in the refugee community?
3. Is a comprehensive case management approach using task sharing to deliver GBV response services acceptable and feasible for improving the health, wellbeing, and safety of GBV survivors in a refugee camp?

### Research methods

**Figure 1.**
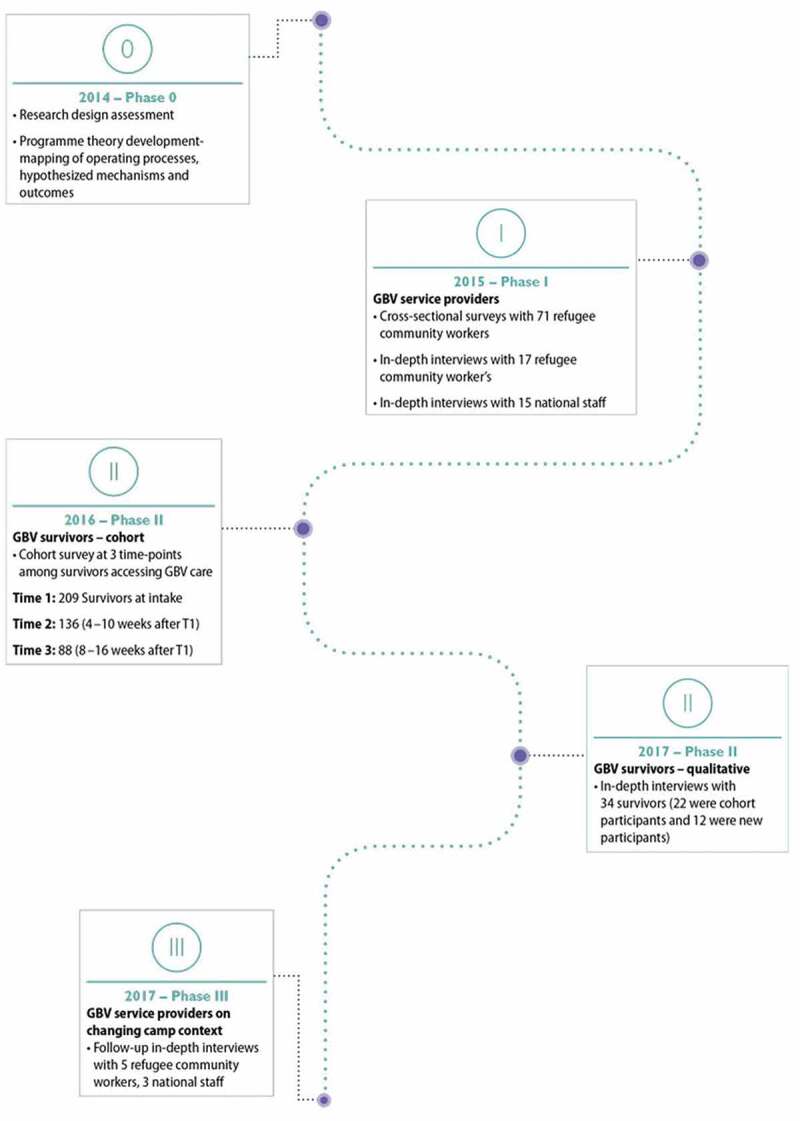
Data collection overview by research phase (Figure from research report [5]).

**Figure 2.**
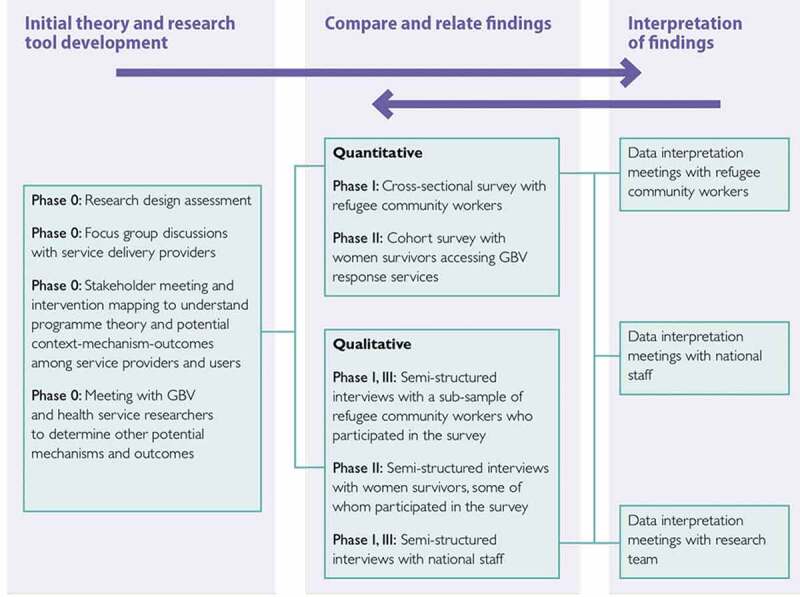
Convergent parallel design and research design components (Figure from research report [5]).

**Figure 3.**
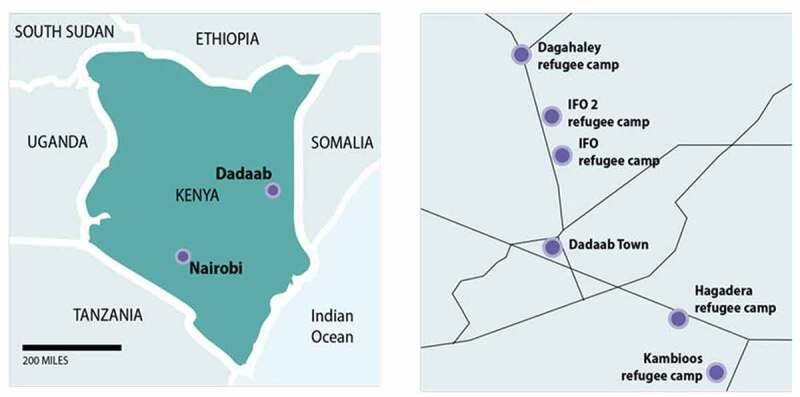
Map of Dadaab complex and individual camps. (Figure from research report [5]).

The research design was a convergent parallel mixed methods evaluation in which both quantitative and qualitative data were triangulated iteratively throughout the study period. This research explored the operations, reach, and delivery of the GBV comprehensive case management intervention by gathering the perspectives of both the recipients and those who were responsible for intervention delivery. Figure 1 outlines the distinct phases of data collection. The study began with a research design assessment phase (i.e. Phase 0) that consisted of focus group discussions and meetings with the IRC and CARE staff, as well as intervention mapping exercises to inform the development of the questionnaires and interview guides. Phase 1 was focused on the experiences of the GBV service providers and included both cross sectional surveys with refugee community workers (n = 71) and in-depth interviews with refugee community workers (n = 17) and national staff (n = 15). Preliminary mixed-methods findings from Phase 1 informed Phase 2 data collection which focused on GBV survivors and consisted of a longitudinal cohort study with three time-periods (T1n = 209, T2n = 136, T3n = 88) and in-depth qualitative interviews with a mix of cohort participants (n = 22) and non-cohort participants (n = 12). Phase 2 mixed-methods preliminary findings, as well as anecdotal feedback we received from Dadaab based staff and researchers, made it clear the study would benefit from adding a final phase (i.e, Phase 3) to conduct follow-up interviews with GBV service providers on the dynamic nature of both GBV prevalence and service provision in the changing camp context. Each phase of data collection was iterative and informed the following phase. Interview questions were adapted to probe further on the emerging relevant themes. Figure 2 outlines the parallel mixed-methods design and Figure 3 provides a map of the fieldwork sites. Table 1 also describes the key elements of the research design. Additional methodological details can be found in the full study report [5] and peer-reviewed papers [6,7], including more detailed accounts of the qualitative data collection and analysis.

**Table 1.** The key research design elements of this GBV comprehensive case-management with task sharing approach.

Research design:	Key elements of this study
**Study site**	Dadaab refugee complex: Hagadera and Dagahaley camps (See Figure 3)
Programme Partners	Two international non-government organisations: International Rescue Committee (IRC) Kenya and Care International Kenya (CARE)
Research Design	Longitudinal mixed-methods design with four data collection phases: **Phase 0**: Research design assessment and programme theory development-mapping **Phase 1**: Cross-sectional survey with refugee community workers (planned n = 75; final n = 71) and in-depth interviews with refugee community workers (n = 17) and national staff (n = 15). **Phase 2**: Longitudinal cohort survey (planned n = 400 GBV survivors at 3 time-points) (final Baseline n = 209; Time 2: 136; Time3: 88; In-depth interviews with GBV survivors (n = 34) **Phase 3**: Additional qualitative interviews with national staff and refugee community workers at IRC-Dadaab to explore the changes in the camp context since Phase 1. (Phase 3 was not in the original research design)
Study sample	**(1) Women survivors of GBV**: 18+ years old and emancipated adolescent girl minors (15–18 years old) accessing GBV response services between 23 February and 23 November 2016 **(2) INGO national staff**: IRC and CARE national staff employed at the time of the study **(3) Refugee community workers**: IRC and CARE recruited community workers
Sampling Frame	**(1) GBV service providers**: The sampling frame was comprised of all current national staff and refugee community workers involved in GBV outreach, community mobilisation, and response delivery. **(2) GBV survivors**: A consecutive sample of eligible women accessing GBV services was invited to participate in the quantitative cohort survey and qualitative in-depth interviews in Phase 2. Qualitative in-depth interview selection was based on various criteria including age, education, marital status, and length of stay in the camp, to ensure a range of perspectives.
Data Collection	**Researchers**: Somali-English speaking female and male interviewers trained and sensitised to the interview topics. We always coordinated same sex interviewers for the refugee community worker and survivor interviews. **Ethical procedures**: informed consent; private, safe interview space; referral to services; confidentiality; same sex interviewers **Data collection tools**: focus group discussion topic guides; intervention mapping tool; cross-sectional survey questionnaire; cohort survey questionnaire; semi-structured interview guides **Interview equipment**: tablet-based surveys; ODK software programmed questionnaires; audio recorded in-depth interviews; ODK encrypted data transfer to LSHTM secure servers

## Expected and unexpected challenges

We expected to encounter a number of research challenges due to a degree of unpredictability and uncertainty for service delivery and research activity in the refugee context. As existing guidelines highlight, the refugee camp setting presents obstacles to implementing sampling frames, ensuring the safety of research participants and the research team, coordinating interview logistics, conducting follow-up interviews, and maintaining high ethical standards [3,4]. Furthermore, because this was an evaluation of an intervention implemented by refugee community workers, the research challenges mirrored the challenging realities that the community workers faced First, we will detail anticipated contextual challenges, the strategies to counteract these challenges, and lessons learned before addressing how we responded to unexpected challenges.

### Anticipated contextual challenge 1. Refugee camps are highly securitized settings

Humanitarian governing bodies assessed the Dadaab refugee complex to be a high-risk setting for the duration of the research period. This limited our access to the residential parts of the complex and prohibited international researchers from staying for extended periods, even in the United Nations (UN) compounds. To address these security risks and our limited access to the study site, the research team implemented a number of strategies.

#### How we responded to this challenge

- Research team members located in Dadaab, Nairobi and London continuously monitored the security risks. IRC and CARE’s Kenya-based security teams gave instruction on when and in what capacity research activities were permitted to ensure safety for the researchers and research participants.

- Due to government restrictions, all research team members needed to have the appropriate Kenyan government identification documents to live and work in Dadaab compounds for extended periods. This meant we were unable to recruit researchers from Somalia which limited field research candidates to Somali-speaking Kenyans. As LSHTM and APHRC sought to hire researchers who could legally work and stay in Dadaab for an extended period without accompaniment of family or a translator, our potential researcher pool tended to be younger Kenyan-Somali who were recent university graduates and often with less experience in conducting empirical data collection. However, to ensure high quality data collection, LSHTM and APHRC led an extended research training and provided on-going support which included regular check-ins and refresher training as needed based on continuous review of the data quality during data collection.

- APHRC and IRC hired a full-time research coordinator to be based at the IRC-Dadaab compound to offer practical support for the research activities and team leadership. The research coordinator ensured that Nairobi and London based research team visits did not disrupt ongoing research and service provision. Having a research coordinator embedded within IRC’s programme helped facilitate relationships between the research team and the refugee community workers, which was instrumental in the implementation and monitoring of research activities.

- **We learned** that receiving approval to enter or reside in the Dadaab compounds was a lengthy bureaucratic process, even when working in close partnership with humanitarian agencies in situ. Additional time should be built into the timeline for these administrative processes, including a potentially lengthy research team recruitment process, to prevent delays and disruptions to research partners and donors.

- **We learned** that the timeline also needed to be flexible because permission to initiate and continue certain activities was dependent on the authorisation of camp-based partners as well.

- **We learned** that the right to work in a refugee camp context should be carefully checked before training and hiring any research team staff.

### Anticipated contextual challenge 2. Refugee camps are hardship locations for researchers

Living and working in the Dadaab setting required the two researchers to live full-time in a humanitarian agency compound separately in two different camps with restricted freedom of movement and access to certain amenities. These restrictions were necessary in order to ensure their safety. The longitudinal design meant that researchers needed to remain in Dadaab for a 6-9-month period facing a range of difficulties and discomforts.

#### How we responded to this challenge

- We conducted an extended two-week training in Nairobi for a larger group of researchers than we needed to be based in Dadaab. This was a paid training and allowed all attendees (i.e. the potential candidates for the researcher positions) and our research team to discern if the work was a good personal and professional fit for the attendees. The majority of the candidates had recently completed a Bachelors or certificate degree. We used this opportunity to ensure the candidates not only met the technical qualifications but also understood what the role and research site would entail. In addition, the researchers who were not selected to go to Dadaab were kept on a reserve list if the selected team member was unable to complete the fieldwork.

- APHRC conducted targeted recruitment through known networks of Somali speaking researchers based in Nairobi. All potential researchers took part in a two-week long training in GBV research and data collection. The training topics ranged from GBV definitions, forms, context, and service delivery options in Dadaab, to research ethics and good interviewing skills. The training was conducted by lead researchers from LSHTM and APHRC and Dadaab field staff providing GBV comprehensive care. The training included an overview of the expected challenges, both research relevant and personal, and the sources of support that would be available to them. This training was an opportunity to facilitate capacity strengthening with the researchers and to ensure higher quality data from the study. As mentioned previously, certain nationality and documentation restrictions limited our hiring pool. However, we were fortunate to identify candidates highly motivated to learn about research methods, but also interested in humanitarian-based research. While the Dadaab-setting restrictions could have complicated hiring a highly qualified team, our research partners invested the time into conducting a supportive training environment, which was successful due to the participation of highly enthusiastic early-career researchers.

- The collaborating research institutions, LSHTM and APHRC, partnered closely with IRC and CARE to plan the researchers’ stay in the camp. We ensured key support people were available including the full-time research coordinator based within the IRC-Dadaab compound, and secure housing. A representative from each of the individual institutions in the research team accompanied the two Dadaab-based researchers on the first trip to ensure a smooth transition into the location and role. The team also went together to meet the refugee community workers and to engage and involve them in the aim of the research.

- We also built in regular rest and recreation leave (R&R) for the study site researchers to return home to visit family and other familiar contexts.

- Nairobi-based team members initiated regular phone check-ins, at a minimum of once per week, with the Dadaab-based researchers to check on their health and wellbeing, with regular messaging between phone calls. A senior research team member in Dadaab or Nairobi was always available if assistance was needed and these needs were then relayed to the London-based team members immediately.

- Senior team members re-informed the Dadaab-based researchers of the around-the-clock support available to them, both in Dadaab through IRC and CARE and at a distance through APHRC and LSHTM, on a regular basis. We facilitated communication between the two study site researchers to encourage shared learning and support as they were based in two separate compound areas for the data collection.

- **We learned** that the regular R&R breaks were critical to the researchers’ long-term wellbeing and the sustainability of the role.

- **We learned** that it was important to critically assess the timeline of the research on a regular basis to determine any need for extensions. This was necessary, not only for the logistics of the research, but to prepare the Dadaab-based researchers for an extended and challenging period of work. Additionally, in the event that they could not stay for a longer period, back-up researchers would need to be recruited.

- **We learned** that it was crucial to maintain a shortlist of potential researchers as a back-up plan. In our case, we maintained the shortlist from the two-week training. The additional back-up researchers could be contacted at times when replacements were needed and, in fact, were needed during the data collection period due to unforeseen leave required by one of our researchers. Having a short list of reserve researchers prevented significant delays in data collection or lost interviews as we were able to quickly hire and provide the new researcher with a refresher training.

### Anticipated contextual challenge 3. Refugee camps are often in remote locations

Reaching Dadaab was only possible via a UN chartered flight which only flew three times each week with limited person-capacity. This required careful planning in advance and only making necessary trips, given the intensive logistics in planning.

#### How we responded to this challenge

- We set up the data collection software for encrypted upload directly to the secure LSHTM server in London, which allowed regular remote data monitoring and cleaning.

- We identified and contracted Somali translation services through an open-call and referrals before the start of the data collection. We used the two-week Nairobi training as an opportunity to assess the translators’ fluencies and proficiencies through quality assessment conducted by Somali-speaking colleagues. We also had their initial translated work checked by Somali-speaking colleagues. We prepared for rolling translation of interviews since it was less feasible to undertake daily recaps and check-ins between the researchers and research managers. Regular transcript reviews and feedback on the quality of interview data served as an alternative to daily research team meetings during data collection.

- **We learned** that because our senior team members were in London and Nairobi, and a fair distance from the study site and the Dadaab-based researchers, we needed to be diligent in providing detailed written feedback. This ensured methodological issues, such as missing data or delayed follow-up interviews, were addressed in a timely manner, enhanced research team members’ confidence, and, importantly, ensured the collection of high-quality data. Written feedback was always followed with a phone call from a Nairobi-based team member to discuss the written feedback and offer further support and mentoring. Some limitations in remote monitoring and feedback meant that these capacity strengthening messages needed to be reinforced as repeated reminders as the researchers were already carrying a large workload in a demanding setting.

### Unexpected challenges

In addition to these lessons, we encountered unanticipated barriers and delays that we had to adapt to as events unfolded. We intended to recruit a sample of 400 refugee women accessing GBV services, which was estimated based on the initial phase of programme mapping and previous programme monitoring data. The longitudinal design required interviews with women at three time points over the course of receiving GBV services. Therefore, it was critical that we were able to maintain contact with the interviewees over a period of 8–12 weeks. Barriers that prevented the research team from accessing residential areas of the camp, combined with the demands on refugees, such as regular food ration collection processes and maintaining homes in a hardship location, made recruitment and follow-up tasks difficult. However, while we anticipated some loss to follow-up, camp-wide policy changes implemented during the data collection period exacerbated these challenges. The primary policy change was that the Kenyan Government announced in May 2016 that the Dadaab refugee complex would close despite ongoing insecurity and dangers in Somalia. This announcement caused acute anxiety and uncertainty amongst the refugee population, who feared forced repatriation would begin imminently. The plan to close Dadaab was followed by various administrative announcements and procedures such as mandatory verification activities, which required refugees to present to the United Nations High Commissioner for Refugees (UNHCR) office for processing. These activities were announced and implemented at short notice with little instruction to camp residents, which resulted in chaotic lines, crowding and long waiting periods at administrative sites. Additionally, our research manager and the IRC and CARE GBV service providers in Dadaab reported to LSHTM and APHRC that these announcements consumed the thoughts of those living in the camp. Survivors explained in interviews or appointment reminder calls that they were less inclined to return for their follow-up appointments at the GBV centres or their follow-up research interviews since they had other administrative appointments and stresses related to the camp closure.

**Figure 4.**
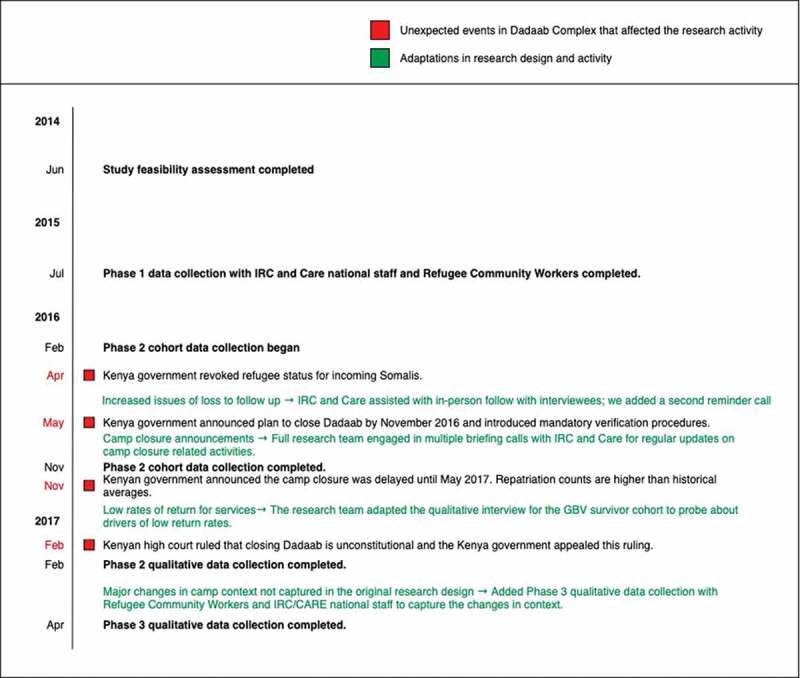
Summary research timeline including unexpected events and research team’s adaptive responses.

These events directly coincided with the longitudinal cohort phase of the data collection activities (Phase 2). This hindered our ability to recruit the anticipated sample size and increased our loss to follow-up among the women accessing GBV services. Figure 4 presents an overview of key events and dates related to the camp-closure announcement and how they coincided with activities in the research timeline.

#### How we responded

We prioritised reducing loss to follow-up. We added a second reminder phone call between the interviews. We also recruited the help of the refugee community workers to follow-up with women in the residential areas, as they had regular and unrestricted contact with the women in the residential areas. This proved to be particularly successful in cases where women were either not responding to phone calls or did not attend their scheduled second or third interview. These delays did, in some cases, lead to longer than expected lapses between interviews.

Upon learning of the camp-closure announcement through our programme partners, Dadaab-based researchers, and local and international news sources, we discussed how to mitigate the loss to follow-up and adapt the research design to account for the smaller sample size. We were able to monitor delays in follow-up during our weekly data monitoring and cleaning. We observed that the follow-up periods were getting longer or being missed entirely and that interviews with new participants were becoming less frequent. This was key to enabling us to identify the issue early and adapt our procedures.

During regular calls with the research team members from LSHTM, APHRC, IRC-Kenya, CARE-Kenya and IRC-UK to discuss the camp events, it quickly became apparent that our discussions were rich with anecdotes from the Dadaab-based research team members. What we learned from these accounts were relevant to the research questions which aimed to understand the context and service delivery in a refugee camp setting. Issues that arose included the challenges that refugee community workers faced while doing their GBV intervention work, such as GBV survivors not having time to meet with them due to attending verification activities, challenges in facilitating outreach activities on the camp-level amidst the upheaval, assisting in following-up with interviewees, as well as some of the personal challenges they faced as both GBV staff and refugees. The task-sharing approach is the key innovative element of this GBV comprehensive case management intervention. Therefore, any challenges the refugee community workers encountered had potential to weaken the structure of the programme. These regular discussions not only helped to improve the data collection procedures, but also indicated the opportunity to add a Phase 3 to the data collection, and later helped inform our data analysis strategy and interpretation of findings.

Particular challenges confront research in humanitarian settings with often unanticipated potential to impact all stages of the research. Utilising a reflexive design and a research team and partners who were able to adapt to dynamic context allowed this study to achieve its research goals. We documented, conferred with each other, analysed incoming data, and adapted the design to capture contextual factors that had an impact on the research and service delivery. Although we originally planned for only one round of qualitative data collection with GBV programming staff, to ensure that we had data describing the changes and its effects on the programming, we conducted an additional round of qualitative interviews with the refugee community workers and national staff (Phase 3). We also adapted the qualitative interview guide for interviews with GBV survivors to include questions about the current events in the camp. The aim of these additional questions was to capture how the camp closure announcements and verification activities affected the lives of the refugee camp residents and the service providers, the overall sentiments and feelings within Dadaab and the research activities. We also asked about ways in which violence in the camp might have changed in response to these camp events. We documented the challenges and allowed them to inform rather than hinder the research. It is important to note that responding to these changes in the camp and capturing the dynamics in the data collected by both adapting interview guides and adding an additional phase of qualitative data collection required additional investments of time and resources (e.g. additional fieldwork travel and logistical costs, interviewers’ time, analysis time, etc.).

Our interviews uncovered that the events surrounding the camp closure announcements created a state of anxiety for many, particularly amongst those who felt that it was unsafe to return to Somalia. Women who decided to return, often under great pressure, reported that they were receiving threats of further violence upon their return to Somalia. Additionally, women’s participation in the verification exercise hindered their ability to attend subsequent research interviews due to time pressures. Strategies we employed to encourage women to return for follow-up interviews included maintaining regular communication with programme partners and study site researchers regarding camp activities, and adapting the data collection tools accordingly; continuing to work with the refugee community workers to follow-up with study participants; and regular monitoring of data to catch and respond to unexpected trends.

In addition to adding Phase 3 and continuing ongoing discussions with the research team, we conducted two formal validation sessions after Phase 1 and Phase 2 were complete. These validation activities entailed a representative from one of the research institution partners presenting the preliminary analysis for critical feedback and open dialogue with the refugee community workers and national staff on the interpretation and potential application of the preliminary findings. These validation sessions resulted in lively discussions and key insights that were integral in guiding the subsequent data collection phases and analysis stage. We recommend that this form of participatory validation with key stakeholder groups should be built into the research timeline as best practice.

## The impacts of these challenges on the data analysis

The cohort questionnaires, administered at three separate timepoints to capture changes over time, provided a breadth of rich information covering several domains (demographics, family, physical and mental health, norms and rights, GBV and exposure to the intervention). However, robust statistical analysis of this data was impacted by the challenges described in this paper, both by the small sample size and the considerable loss-to-follow-up. Despite efforts to limit attrition, only 85 out of 209 (41%) women were able to complete all three cohort interviews. While it is likely that announcement of the camp closure was largely responsible for the high loss-to-follow-up, it is also possible that women who experienced further acts of GBV or worsening physical or mental health were less likely to return for the follow-up interviews. Similarly, it is possible that some women felt they no longer wanted or needed the services. Here, qualitative data helped to triangulate findings and guide the conceptual framework and statistical data analysis strategy. Data from the follow-up interviews were used cautiously to provide insights on service use, particularly how refugee community workers were utilised by participants. However, due to the final sample size much of the analysis focused on the data collected at the baseline interview, at intake to the GBV services.

Another limiting factor was the unknown impact of selection bias on research findings.

To reduce the impact on workload for staff and on service provision, screening was conducted by staff at each centre and they did not document why some women were not selected or did not provide informed consent. Therefore, we are unable to comment on how study participants differ from other women attending the GBV services who did not participate in the study. In particular, there appeared to be systematic differences between participants from Dagahaley and Hagadera in terms of age, income, literacy, and length of encampment with one camp tending to recruit refugees who were slightly older, had higher incomes and more settled compared to another. They also differed considerably by experience of GBV and mental health symptomatic scores with one camp enrolling participants with less severe health outcomes. However, this may be due to differences in demographic characteristics of the camp populations. As this study did not aim to evaluate the impact of service provision on health outcomes, we explored ways to balance gathering data on participant selection processes and the impact of research activities on services and their users.

As an innovative service model, this study was designed to understand factors that could strengthen GBV services provided by humanitarian agencies in Dadaab and other humanitarian contexts. We were unable to design a study that allowed for a control group due to practical and ethical constraints. In settings where it is not possible to build in a control group for service evaluation, other study designs could be explored such as self-controlled case series and cluster-randomised control trials. In Dadaab it was not feasible to modify or standardise the comprehensive case management model for different survivors or deny survivors access to any needed services. Therefore, the lack of a suitable control group meant that we could not isolate the effect of the GBV services on core survivor outcomes (such as mental health) over time although we were able to examine trends over time. Similarly, we were unable to establish the direct impact of task-sharing on the delivery of this case management intervention. Free-text fields within the quantitative survey and qualitative data helped to mitigate the impact of this limitation.

Despite these limitations, this research afforded rare insights into experiences of GBV in conflict-affected settings, its consequences on health and wellbeing, and the use of GBV case-management services within Dadaab. Ultimately, the challenges imposed by the repatriation process impacted the external validity of the findings and their generalisability to refugee women experiencing GBV in other camp settings. This study aimed to understand the process, feasibility and acceptability of GBV response services in a camp setting. The data collection process ultimately allowed us to capture some of the consequences of a common humanitarian setting process – repatriation – on women’s access to and use of GBV response services. The repatriation process had the unintended consequence of limiting GBV survivors’ access to needed comprehensive care response services.

## Conclusion

Humanitarian settings are dynamic places and researchers must adopt a reflexive design to capture the many emerging states of a crisis that can occur during data collection. Conducting research in humanitarian settings requires a flexible approach to accommodate these unexpected challenges that can impact both service delivery and research activities. This will often require longer study periods and additional flexible budgets to cover delays or unexpected additional data collection periods to capture any fundamental changes in the context. These investments are necessary to understand the barriers to delivering critical services in humanitarian settings, such as GBV prevention and response. The inclusion of an additional qualitative phase improved our understanding of the task-sharing intervention. Without it, our interpretation of the data collected in this context would be far more limited. The unexpected smaller sample size, the high rates of loss to follow-up and the delays in follow-up are not solely an absence of data or limitation of the research, they are in themselves a statement of what was taking place in Dadaab and how service provision was impacted.

Our study shows that GBV research in refugee camps is challenging but feasible. We recommend that research partnerships working in these settings, including donors, initiate discussions early and regularly about how to keep the research design flexible, relevant to the changing context, inclusive of multiple perspectives from the target population at both research design and analysis stages and aware of the time, human and financial resources this might require. It is important that our research gives a full, accurate and critical account that reflects the reality of a humanitarian setting.

## Paper context

There are many methodological, ethical and logistical challenges to research in humanitarian settings, especially on sensitive topics such as gender-based violence. These challenges are broadly covered by humanitarian research guidelines, but there are few in-depth methodological case studies from recent studies. This methods forum contributes in-depth reflections on the challenges and implemented solutions while conducting a mixed methods evaluation of a gender-based violence intervention in a refugee-camp setting to share detailed methodological lessons more widely.
